# Editorial: Women in psychiatry 2023: psychopharmacology

**DOI:** 10.3389/fpsyt.2024.1488260

**Published:** 2024-11-12

**Authors:** Rosana Camarini, Alfreda Stadlin, Reem Kais Jan

**Affiliations:** ^1^ Department of Pharmacology, Institute of Biomedical Sciences, University of São Paulo, São Paulo, Brazil; ^2^ College of Medicine, Ajman University, Ajman, United Arab Emirates; ^3^ College of Medicine, Mohammed Bin Rashid University of Medicine and Health Sciences, Dubai, United Arab Emirates

**Keywords:** psychiatric disorder, eating disoders, substance use, addiction, computerized physician order, schizophrenia, ethanol sensitization

The advancement of scientific knowledge is often propelled by the innovative work of dedicated researchers. The third edition of “*Women in Psychiatry 2023: Psychopharmacology*” highlights six articles led by women scientists, demonstrating their contributions to understanding psychiatric/eating disorders, substance/alcohol use disorder, and pharmacological interventions. These studies not only advance our scientific understanding but also underscore the importance of female leadership in research, driving forward critical investigations into mental health and addiction. Through their exemplary work, these women inspire the next generation of scientists, fostering a more inclusive and diverse research community that enriches the field of psychiatry.

## Psychiatric and eating disorders

Patients with 22q11.2 deletion syndrome face a high risk of psychiatric morbidity, underscoring the importance of monitoring and mitigating the long-term impact of psychopathology in these individuals (1). In “*Effects of risperidone on psychotic symptoms and cognitive functions in 22q11.2 deletion syndrome: Results from a clinical trial*”, Latrèche et al. conducted a double-blind randomized controlled trial in carriers of the 22q11.2 deletion syndrome to investigate the neuroprotective effects of the antipsychotic risperidone, administered during adolescence. The treated group exhibited greater short-term improvements in psychotic symptoms, and preliminary results indicated that early antipsychotic treatment may reduce or prevent long-term deteriorations in clinical symptoms and cognitive skills, particularly in working memory and attention.

Eating disorders (ED) affect 1-2% of adolescents, leading to significant morbidity and mortality, with onset typically occurring during adolescence. ED involves compulsive behaviors, such as binge eating or purging, which are influenced by the opioid system and affect food palatability and pleasure (2). Naltrexone, an opioid antagonist, is used to mitigate these behaviors, though its efficacy and optimal dosage remain uncertain. In “*Potential biomarker of brain response to opioid antagonism in adolescents with eating disorders: a pilot study*,” Stancil et al. explored the use of functional magnetic resonance imaging (fMRI) as a pharmacodynamic biomarker to detect brain response to naltrexone in adolescents with binge/purge ED. Results indicated significant changes in blood oxygenation-level dependent (BOLD) signals in reward-related brain regions, specifically the nucleus accumbens during passive food viewing and the anterior cingulate cortex during the monetary incentive delay task, suggesting that fMRI could serve as a valuable tool in optimizing naltrexone therapy and developing novel treatments targeting the reward pathway in ED.

## Substance use and addiction

The high prevalence of e-cigarette use (5) and the frequent prescription of fluoxetine during adolescence (6) underscore the need for further research into their combined effects on the developing brain. A study by Yuan and Leslie titled “*Nicotine and fluoxetine alter adolescent dopamine-mediated behaviors via 5-HT_1A_ receptor activation*” found that nicotine and fluoxetine exposure in adolescent rats enhanced responses to dopaminergic drugs and increased initial cocaine self-administration. These effects were mediated by serotonin 5-HT1A receptors, suggesting that maladaptive changes in 5-HT signaling may prime adolescents for future substance abuse. This research highlights the need for targeted interventions during adolescence to prevent long-term substance use disorders.

Ethanol-induced sensitization (7) and tolerance (8) are complex phenomena with potential genetic and environmental influences. The study by Reed and Phillips Richards “*Does tolerance to ethanol-induced ataxia explain the sensitized response to ethanol?*” used diverse mouse strains and genetically heterogeneous stock to assess the relationship between these phenomena, measuring changes in activity and coordination. Results indicated significant individual variability and a partial correlation between sensitization and tolerance, particularly in genetically heterogeneous populations. The genetic factors that determine the degree of sensitization and tolerance seem to operate independently in these mouse strains. The findings suggest that while genetic mechanisms may not be shared, environmental factors play a crucial role in these behaviors. Therefore, both sensitization and tolerance should be regarded as independent processes, each potentially influencing susceptibility to addiction.

The 2023 World Drug Report highlights a significant rise in global amphetamine-type stimulant (ATS) use, including methamphetamine, with 36 million users in 2021. Notably, 45% of current ATS users are women, yet only 25% receive treatment, despite women progressing through addiction more rapidly than men (3). In this context, Fultz et al. in “*Effects of systemic pretreatment with the NAALADase inhibitor 2-PMPA on oral methamphetamine reinforcement in C57BL/6J mice*” investigated the effects of 2-PMPA on oral methamphetamine (MA) reinforcement in mice. Their study found that while a high dose of 2-PMPA (100 mg/kg) reduced certain behaviors associates with MA self-administration in male and female mice, a lower dose (30 mg/kg) did not, and overall drug intake remained unaffected. These results underscore the complexity of MA addiction and the necessity for further research to assess 2-PMPA’s efficacy and to explore additional glutamate-targeting strategies.

## Pharmacological interventions

The study “*Reduced prevalence of drug-related problems in psychiatric inpatients after implementation of a pharmacist-supported computerized physician order entry system - a retrospective cohort study*” by Wien et al. aimed to investigate whether the implementation of a computerized physician order entry (CPOE) system with an integrated clinical decision support system (CDSS) at a psychiatric hospital in Lübeck, Germany, reduced drug-related problems (DRPs). Psychiatric patients often experience complex polypharmacy of psychopharmacological agents (4). High rates of anticholinergic drug use contribute to significant clinical risks and adverse drug events. Results showed a significant reduction in DRPs, particularly prescription errors, and fewer unresolved DRPs at discharge, highlighting the system’s efficacy in improving medication safety.

## Conclusion

The studies reviewed in this editorial offer valuable insights into psychotic disorders, eating disorders, substance use, and pharmacological interventions. From early intervention in high-risk populations, such as those with 22q11.2 deletion syndrome, to optimizing treatments for EDs, improving medication safety in psychiatric settings, exploring new addiction therapies, and understanding the complexities of sensitization and tolerance, these findings contribute significantly to future research and clinical advancements.
